# 

**Published:** 1900-02-17

**Authors:** 


					THE HOSriTAL. "The standard of highest purity.'1?l.anr.ei. Fkbrcary 1/th, 1900. .
CADc?o?RYS fH|
ABSOLUTELY PURR.
NO DRUGS OR ALKALIES USED.
No. 699. ' i Vol. XXVII. [JffiSS&H Saturday,
Feb. 17, 1900.
"Sui scription Terms,] pE(ICE TWOPENCE.

				

